# Relationship of Fibroblast Growth Factor 23 Serum Levels with Disease Characteristics in Systemic Lupus Erythematosus Patients

**DOI:** 10.3390/biom13081222

**Published:** 2023-08-05

**Authors:** Yolanda Fernández-Cladera, Fuensanta Gómez-Bernal, María García-González, Juan C. Quevedo-Abeledo, Agustín F. González-Rivero, Antonia de Vera-González, Candelaria Martín-González, Ana L. Nunes-Andrade, Raquel López-Mejías, Miguel Á. González-Gay, Iván Ferraz-Amaro

**Affiliations:** 1 Division of Central Laboratory, Hospital Universitario de Canarias, 38320 Tenerife, Spain; yolanda.fernandezcladera@gmail.com (Y.F.-C.); fuensanta95@gmail.com (F.G.-B.); afgonriv@gmail.com (A.F.G.-R.); adeverag@gmail.com (A.d.V.-G.); 2 Division of Rheumatology, Hospital Universitario de Canarias, 38320 Tenerife, Spain; margagon23@hotmail.com; 3 Division of Rheumatology, Hospital Doctor Negrín, 35010 Las Palmas de Gran Canaria, Spain; quevedojcarlos@yahoo.es; 4 Division of Internal Medicine, Hospital Universitario de Canarias, 38320 Tenerife, Spain; mmartgon@ull.edu.es; 5 Department of Internal Medicine, University of La Laguna (ULL), 38200 Tenerife, Spain; alu0101155869@ull.edu.es; 6 Epidemiology, Genetics and Atherosclerosis Research Group on Systemic Inflammatory Diseases, Instituto de Investigación Sanitaria Marqués de Valdecilla (IDIVAL), 39011 Santander, Spain; rlopezmejias78@gmail.com; 7 Division of Rheumatology, IIS-Fundación Jiménez Díaz, 28015 Madrid, Spain; 8 Department of Medicine, University of Cantabria, 39011 Santander, Spain; 9 Cardiovascular Pathophysiology and Genomics Research Unit, School of Physiology, Faculty of Health Sciences, University of the Witwatersrand, Johannesburg 2000, South Africa

**Keywords:** fibroblast growth factor 23, systemic lupus erythematosus, disease damage, musculoskeletal complications

## Abstract

Fibroblast growth factor 23 (FGF23), a hormone secreted by osteocytes and osteoblasts, is a major regulator of vitamin D and phosphate homeostasis. FGF23 has been associated with the disturbance of mineral homeostasis, and with kidney and cardiovascular diseases. Systemic lupus erythematosus (SLE) is an autoimmune disorder that can affect virtually any organ. In the present work, we set out to analyze the relationship of FGF23 with the expression of SLE, including patterns of activity, damage, and severity. A total of 284 well-characterized patients with SLE were recruited. Activity (SLEDAI), severity (Katz), and damage index (SLICC-DI) scores were determined. The serum levels of FGF23 were also assessed. Multivariable linear regression analysis was performed to study the relationship between disease characteristics and FGF23. FGF23 and 25(OH) vitamin D were negatively correlated. Furthermore, prednisone use was associated with higher circulating FGF23 after an adjustment for confounding factors. SLICC-DI was related to higher serum levels of FGF23 after a multivariable analysis. However, when the SLICC-DI index items and domains were analyzed separately, apart from proteinuria ≥3.5 gm/24 h, only the musculoskeletal domain, encompassing arthritis and osteoporosis, was significantly associated with higher serum levels of FGF23. In conclusion, an association is observed between elevated serum FGF23 levels and disease damage, particularly related to musculoskeletal complications and proteinuria, in patients with SLE.

## 1. Introduction

Fibroblast growth factor 23 (FGF23) is a circulating peptide that plays a key role in the control of serum phosphate concentrations. FGF23 is secreted by bone osteocytes and osteoblasts in response to calcitriol, increased dietary phosphate load, parathormone, and calcium [1]. The main function of FGF23 is to maintain a normal serum phosphate concentration by reducing renal phosphate reabsorption and intestinal phosphate absorption through decreased calcitriol production. In renal proximal tubular cells, FGF23 binds to the FGF receptor and its coreceptor, klotho [2]. α-Klotho enhances the binding affinity of FGF23 for the FGF receptor and confers tissue specificity to the action of FGF23 due to its limited expression, predominantly in the kidneys and parathyroid glands. FGF23 measurement has been advocated as an early and sensitive marker for chronic kidney disease-related bone disease [3]. Moreover, high FGF-23 levels have been associated with multiple adverse cardiovascular outcomes, including hypertension, left ventricular hypertrophy, subclinical atherosclerosis and cardiovascular events, and mortality [4].

Systemic lupus erythematosus (SLE) is a chronic autoimmune disease characterized by immune system dysfunction and is clinically heterogeneous, exhibiting, among others, renal, dermatological, neuropsychiatric, and cardiovascular manifestations. The clinical heterogeneity of SLE and the lack of pathognomonic biomarkers pose a diagnostic challenge for the clinician [5]. For this reason, robust immunological biomarkers are needed to better understand disease diagnosis and progression in individuals with SLE, including non-organ-specific and organ-specific SLE biomarkers [6].

In the present work, we evaluate FGF23 in a large series of well-characterized patients with SLE. Our objective is to analyze the relationship of FGF23 with disease-related features, including disease activity, damage, and severity, as well as with subclinical atherosclerosis accompanying SLE.

## 2. Materials and Methods

### 2.1. Study Participants

This was a cross-sectional study that included 284 patients with SLE. All patients with SLE were 18 years old or older, had a clinical diagnosis of SLE, and met ≥4 American College of Rheumatology (ACR) classification criteria for SLE [7]. They were diagnosed by rheumatologists and were regularly followed-up in rheumatology outpatient clinics. Participation was allowed for patients taking prednisone, at an equivalent dose of ≤10 mg/day, as glucocorticoids are often used in the treatment of SLE. The research adhered to the principles outlined in the Declaration of Helsinki. The study protocol received approval from the Institutional Review Committee at Hospital Universitario de Canarias and Hospital Universitario Doctor Negrín, both located in Spain. All participants provided informed written consent before participating in the study (Approval Number 2015_84).

### 2.2. Data Collection and Laboratory Assessments

Participants in the study completed a questionnaire on cardiovascular risk factors and medication use, and also underwent a physical examination. The examination included measurements of weight, height, body mass index, abdominal circumference, as well as systolic and diastolic blood pressure levels (measured while the participant was in a supine position). These measurements were taken under standardized conditions. Smoking status and hypertension treatment information were obtained from the questionnaire. In addition, medical records were reviewed to gather information on specific diagnoses and medications. The activity and damage related to SLE were evaluated using the Systemic Lupus Erythematosus Disease Activity Index -2000 (SLEDAI-2K) [8] and Systemic Lupus International Collaborating Clinics/American College of Rheumatology (SLICC/ACR Damage Index -DI-) [9], respectively. For the present study proposal, the SLEDAI-2k index was divided into none (0 points), mild (1–5 points), moderate (6–10 points), high (11–19), and very high activity (>20) as described above, as previously described [10]. The severity of the disease was measured using the Katz index [11]. In addition, carotid ultrasonography was performed to assess carotid intima-media wall thickness (cIMT) in the common carotid artery and to identify focal plaques in the extracranial carotid according to Mannheim consensus definitions [12,13]. The 25(OH) vitamin D levels were assessed by chemiluminescence. An enzyme-linked immunosorbent assay—ELISA—kit was used for the detection of FGF23 (Elabscience, Houston, TX, USA). Both intra- and inter-coefficients of variability were <10% for this assay. Cholesterol, triglycerides, and HDL-cholesterol were measured using the enzymatic colorimetric assay (Roche, Basel, Switzerland). Lipoprotein A and lipoproteins were assessed using a quantitative immunoturbidimetric assay (Roche). Phosphate was assessed through the spectrophotometric detection of a colored phosphomolybdate complex. The atherogenic index was calculated using the total cholesterol/HDL-C ratio according to the Castelli formula. LDL cholesterol was calculated using the Friedewald formula. Dyslipidemia was defined if total cholesterol was >200 mg/dL or triglycerides were >150 mg/dL or LDL was > 130 mg/dL or HDL was mg/dL <40 in males or HDL was mg/dL <50 in females.

### 2.3. Statistical Analysis

Demographic and clinical characteristics in patients with SLE and controls were described as mean ± standard deviation (SD) or percentages for categorical variables. For non-normally distributed continuous variables, the data were expressed as median and interquartile range (IQR). The relationship of disease characteristics with circulating FGF23 was evaluated by multivariable linear regression analysis. The normality of variables was tested, and diagnostics of linear regression models were performed studying the residuals plots of the models. Where necessary, the transformation of variables was performed to allow linearity of the models constructed. All the analyses used a 5% two-sided significance level and were performed using Stata software, version 17/SE (StataCorp, College Station, TX, USA).

## 3. Results

### 3.1. Demographics and Disease-Related Data of Systemic Lupus Erythematosus Patients

The median (IQR) serum levels of FGF23 in patients with SLE were 88 (IQR 40–149) pg/mL. Demographic and disease-related characteristics of the 284 SLE patients included in this study are presented in Table 1. The majority of the participants were female (92%), with a mean age ± SD of 50 ± 12 years. The average body mass index was 28 ± 6 kg/m^2^ and the mean abdominal circumference was 92 ± 13 cm. Classic cardiovascular risk factors were relatively common among the patients, with 24% being current smokers, 39% having hypertension, 77% exhibiting dyslipidemia, and 30% being obese. Additionally, 25% of the patients were taking statins and 29% were taking aspirin (Table 1).

The median disease duration was 16 (IQR 7–24) years. The majority of SLE patients had either no disease activity (40%) or mild–moderate activity (39%) according to the SLEDAI score. The SLICC-DI and Katz indices had a median of 1 (IQR 0–2) and 2 (IQR 1–4), respectively. About 78% of the patients had a SLICC-DI score equal to or higher than 1. Half of the patients (50%) were taking prednisone, with a median daily dose of 5 mg/day (IQR 5–7.5 mg). At the time of recruitment, 67% of the patients tested positive for anti-DNA antibodies and 69% for anti-ENA antibodies, with anti-SSA being the most frequently detected antibody (35%). Sixty-nine percent of the patients were taking hydroxychloroquine during the study. Less commonly used disease-modifying antirheumatic drugs included methotrexate (11%) and azathioprine (15%).

In terms of carotid atherosclerosis, the average cIMT was 628 ± 109 microns and 36% of the patients had carotid plaque detected in the ultrasound assessment. Additional information related to the SLE data is provided in Table 1.

### 3.2. Demographic and Disease Characteristics in Relation to Serum FGF23 Levels

**Figure 1:**
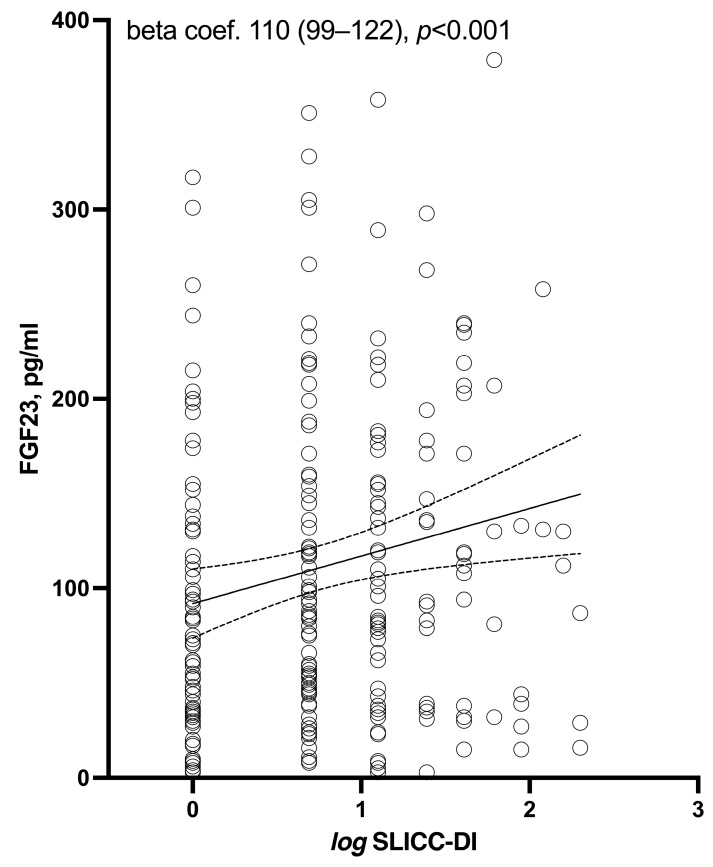
Relation between log SLICC-DI and FGF-23 serum levels.

In the univariable analysis, age, obesity, dyslipidemia, and statin use were significantly associated with higher circulating FGF23 levels (Table 2). Furthermore, serum 25(OH) vitamin D levels, but not circulating phosphate, showed a high and negative association. Regarding disease-related data, serum CRP levels revealed a positive and significant association with FGF23. However, after a multivariable analysis, the significance of this relationship was lost. Katz and SLEDAI scores were not associated with FGF23 in the univariable analysis. This was not the case for SLICC-DI, which, when considered as binary (greater than or equal to 1) and continuous, showed a significant relationship with higher FGF23 levels (Figure 1). Remarkably, this association with SLICC-DI remained significant after the multivariable analysis adjusted by age, smoking, hypertension, obesity, dyslipidemia, and 25(OH) vitamin D serum levels (Table 2). Other disease-related variables that were significantly associated with FGF23 was the use of prednisone. Although cIMT had a significant positive relationship with FGF23 in the univariable analysis, no association was found between carotid plaques and FGF23 (Table 2).

### 3.3. Relationship of Activity Score and Damage and Disease Severity Indices with FGF23

Since the activity score and the damage and disease severity indices are a sum of different aspects of SLE, we show in Table 3 the relationship of each item of these scores with FGF23. Regarding the Katz index, history of cerebritis (seizure or organic brain syndrome) (n = 12) and the lowest recorded hematocrit to date (values < 30%) (n = 47) were, respectively, associated with higher and lower serum levels of FGF23. SLEDAI score items were in general not associated with circulating FGF23. In this regard, only the presence of mucosal ulcers (n = 14) showed a significant association with FGF23. With respect to SLICC-DI items, a musculoskeletal domain value equal to or higher than 1 (n = 89) was the only domain of this score that disclosed a positive and significant relationship with FGF23. A full list of individualized SLICC-DI items is shown in Supplementary Table S1. In this representation of all the SLICC items, proteinuria ≥3.5 g/24 h (n = 7), arthritis (n = 40), and osteoporosis (n = 23) were significantly associated with higher levels of FGF23.

## 4. Discussion

The present study includes a large series of SLE patients specifically tested for FGF23. According to our results, the damage caused by the disease is significantly and independently associated with higher levels of this hormone. Furthermore, disease damage, particularly related to musculoskeletal complications and proteinuria, are significantly associated with FGF23 upregulation in these SLE patients.

There is limited information on this topic in the literature. In this sense, a previous study showed higher levels of FGF23 in 15 premenopausal patients with newly diagnosed lupus nephritis (≤2 months) compared to 15 age-matched healthy controls (12). Furthermore, in this study, FGF23 levels showed a correlation with urinary monocyte chemotactic protein 1, serum TNF-α, and serum IL-6. However, only the correlation between FGF23 and monocyte chemotactic protein 1 remained statistically significant after adjustments for 25(OH) vitamin D and renal function.

In line with the abovementioned points, a case report described a 12-year-old girl with lupus nephritis and an elevated serum level of FGF23. Following mycophenolate mofetil treatment, serum complement levels increased as the FGF23 level decreased (13). Proteinuria is a strong marker for the progression of chronic kidney disease and our finding that patients with proteinuria ≥3.5 g/day exhibit higher levels of FGF23 support these two previous reports and highlight the potential role of FGF23 in the development of chronic kidney disease in patients with SLE. This is in agreement with the data from the general population showing that an increase in FGF23 constitutes one of the first detectable biomarkers of chronic kidney disease [14]. Of note, FGF23 levels are increased in chronic kidney disease even before changes in serum calcium, phosphorous, or PTH levels [15]. In renal proximal tubular cells, FGF23 binds to the FGF receptor and its coreceptor, klotho, causing the downregulation of the luminal membrane sodium phosphate cotransporter. Decreased cotransporters in the proximal tubule lead to reduced phosphate reabsorption and increased urinary phosphate excretion. FGF23 also inhibits the proximal tubular expression of the 1-alpha-hydroxylase enzyme, leading to decreased calcitriol synthesis by the kidney. We believe our findings of a relation between proteinuria and FGF23 are in line with its physiological role.

Glucocorticoids are commonly used in the management of patients with SLE. In this regard, a study focused primarily on bone turnover in 72 SLE patients and 10 age- and sex-matched healthy individuals revealed significantly higher serum levels of FGF23 in patients treated with glucocorticoids than in those not taking them [16]. In contrast, FGF23 did not differ between the controls and patients not taking glucocorticoids. In addition, a negative relationship was found between FGF23 and the serum levels of osteocalcin and 25 (OH) vitamin D. In accordance with these data, a negative relationship between 25 (OH) vitamin D and FGF23 was also found in our study. This is consistent with the physiological role of FGF23, which promotes reduced levels of 25(OH) vitamin D by inhibiting renal α-1 hydroxylase and blocking parathormone synthesis and release. In our cohort, the use of prednisone was associated with higher serum levels of FGF23. However, due to the cross-sectional design of our study, the relationship between prednisone and FGF23 could not be clearly inferred. Perhaps, rather than a direct effect of prednisone on FGF23, this may be due to the fact that prednisone is used more frequently in patients with greater disease damage. This would be consistent with the positive relationship between the SLICC-DI score and FGF23 found in our work. Moreover, except for corticosteroids, we found no relationship between the treatments used for the disease and FGF23. We believe that this may have been due to the cross-sectional design of our study. Furthermore, possibly, the biological mechanisms through which the treatments exert their functions may not be related to this molecule.

In a previous work of our group, α-Klotho protein serum levels did not differ between patients with SLE and controls. Nevertheless, SLE patients taking prednisone or those with musculoskeletal manifestations had significantly higher circulating levels of α-Klotho [17]. These results, together with those shown in the present study with respect to FGF23, may indicate that the α-Klotho–FGF23 axis is altered in patients with SLE, with the musculoskeletal manifestations of the disease probably being the most influenced by the dysregulation of this axis.

We also found a significant positive association between cIMT and FGF23 on the univariable analysis that did not hold after an adjustment for covariates. This may be of potential relevance as it may be consistent with the previously described role of FGF23 in cardiovascular disease in the general population [4]. However, there are no specific reports addressing the role of FGF23 in cardiovascular disease in SLE patients. The fact that FGF23 was related both to disease damage and, albeit univariably, cIMT would support a relationship between FGF23 and cardiovascular disease in patients with SLE.

Interestingly, in our work, the levels of FGF and phosphate did not correlate. This was surprising and unexpected. We do not have an exact explanation for this. Perhaps, in patients with SLE, there is a disruption of this axis caused by the disease itself or by the inflammatory state that the disease exerts.

We recognized the limitation that we did not recruit controls into our work. This could have helped us to know if FGF23 levels differ between healthy and SLE populations. However, our purpose was primarily to study the relationship of this molecule with the manifestations of the disease. Furthermore, although we did not record whether the patients were taking calcium and vitamin D supplements, serum levels of 25(OH) vitamin D in our patients were within normal ranges, ruling out that any vitamin D deficiency could have affected our results. We also acknowledged that having performed multiple comparisons with FGF23 may have caused some associations to occur at random. However, our study was based on the a priori hypothesis that disease characteristics are related to FGF23, and not based on performing multiple comparisons to find a significant one. Another fact to highlight is that many patients were receiving treatment with corticosteroids. The exact mechanisms of how these affect FGF23 deserve further study.

## 5. Conclusions

In conclusion, FGF23 is associated with the damage produced by the disease in patients with SLE. This would imply that FGF23 plays a role in the pathophysiology of the disease, either causally or as a consequence of the immunoinflammatory alteration that occurs in SLE. FGF23 can be considered a biomarker of musculoskeletal and kidney diseases in SLE patients.

## Figures and Tables

**Table 1:** Characteristics of the cohort of SLE patients.

	SLE Patients
	(n = 284)
Age, years	50 ± 12
Female, n (%)	261 (92)
Body mass index, kg/m^2^	28 ± 6
Abdominal circumference, cm	93 ± 14
Hip circumference, cm	103 ± 12
Waist-to-hip ratio	0.90 ± 0.07
FGF23, pg/mL	88 (40–149)
25(OH) vitamin D, ng/mL	31 ± 12
Phosphate, mg/dL	3.5 ± 0.6
Cardiovascular co-morbidity	
Smoking, n (%)	69 (24)
Diabetes, n (%)	16 (6)
Hypertension, n (%)	111 (39)
Obesity, n (%)	85 (30)
Dyslipidemia, n (%)	220 (77)
Statins, n (%)	72 (25)
Aspirin, n (%)	80 (29)
Carotid intima-media thickness, microns	628 ± 109
Carotid plaque, n (%)	99 (36)
SLE-related data	
Disease duration, years	16 (7–24)
CRP, mg/dL	2.0 (0.8–4.4)
SLICC-DI	1 (0–2)
SLICC-DI ≥ 1, n (%)	191 (68)
Katz Index	2 (1–4)
Katz ≥ 3, n (%)	126 (44)
SLEDAI	2 (0–4)
SLEDAI categories, n (%)	
No activity, n (%)	109 (40)
Mild, n (%)	107 (39)
Moderate, n (%)	41 (15)
High, n (%)	10 (4)
Very High, n (%)	4 (1)
Auto-antibody profile	
Anti-DNA positive, n (%)	151 (67)
Anti-ENA positive, n (%)	164 (69)
Anti-SSA, n (%)	55 (35)
Anti-SSB, n (%)	36 (21)
Anti-RNP, n (%)	64 (28)
Anti-Sm, n (%)	24 (10)
Anti-ribosome	13 (9)
Anti-nucleosome	32 (22)
Anti-histone	22 (15)
Antiphospholipid syndrome, n (%)	43 (16)
Antiphospholipid autoantibodies, n (%)	61 (32)
Lupus anticoagulant, n (%)	51 (28)
ACA IgM, n (%)	22 (11)
ACA IgG, n (%)	39 (20)
Anti-beta2 glycoprotein IgM, n (%)	19 (10)
Anti-beta2 glycoprotein IgG, n (%)	28 (15)
C3, mg/dL	130 ± 40
C4, mg/dL	21 ± 12
Current prednisone, n (%)	140 (50)
Prednisone, mg/day	5 (5–7.5)
Hydroxychloroquine, n (%)	194 (69)
Methotrexate, n (%)	31 (11)
Mycophenolate mofetil, n (%)	31 (11)
Azathioprine, n (%)	43 (15)
Rituximab, n (%)	8 (3)
Belimumab, n (%)	8 (3)

Data represent mean ± SD or median (interquartile range) when data were not normally distributed. BMI: body mass index; C: complement; CRP: C-reactive protein; LDL: low-density lipoprotein. ACA: anticardiolipin. HDL: high-density lipoprotein; anti-ENA: extractible nuclear antibodies. SLEDAI: Systemic Lupus Erythematosus Disease Activity Index. SLEDAI categories were defined as: 0, no activity; 1–5 mild; 6–10 moderate; >10 high activity, >20 very high activity. SLICC-DI: Systemic Lupus International Collaborating Clinics/American Colleague of Rheumatology Damage Index. Dyslipidemia is defined if total cholesterol is >200 mg/dL or triglycerides >150 mg/dL or LDL >130 mg/dL or HDL <40 in males or HDL mg/dL <50 in females. FGF23: fibroblast growth factor 23.

**Table 2:** Relationship of demographic and disease characteristics with FGF23 serum levels.

	FGF23, pg/mL			
	Beta Coef. (95%), *p*			
	Univariable		Multivariable	
Age, years	**1.3 (0.3–2)**	**0.013**		
Female	−2 (−46–42)	0.93		
Body mass index, kg/m^2^	1 (1–3)	0.24		
Abdominal circumference, cm	0.7 (−0.2–1.5)	0.13		
Hip circumference, cm	0.3 (−0.7–1)	0.54		
Waist-to-hip ratio	135 (−25–295)	0.098		
25 (OH) vitamin D, ng/mL	**−2 (−4–(−0.6))**	**0.007**		
Phosphate, mg/dL	−2 (−35–31)	0.92		
Cardiovascular co-morbidity				
Smoking	21 (−6–48)	0.12		
Diabetes	12 (−38–61)	0.64		
Hypertension	18 (−5–42)	0.13		
Obesity	**35 (10–60)**	**0.007**		
Dyslipidemia	**32 (5–59)**	**0.022**		
Statins	**41 (15–68)**	**0.002**		
Aspirin	−0.4 (−27–26)	0.98		
SLE-related data				
Disease duration, years	0.6 (−0.6–1.8)	0.33		
CRP, mg/dL	**1 (0.05–2)**	**0.040**	0.5 (−0.8–2)	0.45
log SLICC-DI	**110 (99–122)**	**<0.001**	**35 (2–68)**	**0.036**
SLICC-DI ≥ 1	**34 (10–59)**	**0.007**	**48 (4–92)**	**0.033**
Katz Index	1 (−5–7)	0.81		
Katz ≥ 3	−4 (−28–19)	0.72		
SLEDAI	−0.4 (−3–2)	0.79		
SLEDAI categories				
No activity	ref.	ref.		
Mild	6 (−21–32)	0.68		
Moderate to very high	−8 (−40–24)	0.61		
Auto-antibody profile				
Anti-DNA positive	17 (−8–43)	0.18	35 (−4–75)	0.074
Anti-ENA positive	−9 (−35–17)	0.52		
Anti-SSA	17 (−17–50)	0.32		
Anti-SSB	38 (−36–113)	0.31		
Anti-RNP	−5 (−32–22)	0.72		
Anti-Sm	35 (−4–74)	0.078	26 (−57–110)	0.53
Anti-ribosome	−32 (−89–24)	0.26		
Anti-nucleosome	−26 (−66–15)	0.21		
Anti-histone	1 (−47–48)	0.98		
Antiphospholipid syndrome	11 (−19–40)	0.48		
Antiphospholipid autoantibodies				
Lupus anticoagulant	−11 (−42–20)	0.48		
ACA IgM	19 (−23–60)	0.37		
ACA IgG	2 (−31–36)	0.89		
Anti-beta2 glycoprotein IgM	−11 (−58–36)	0.65		
Anti-beta2 glycoprotein IgG	19 (−20–57)	0.34		
C3, mg/dL	0.05 (−0.3–0.4)	0.75		
C4, mg/dL	0.1 (−0.9–1)	0.81		
Current intake of prednisone	**28 (5–51)**	**0.018**	**40 (1–79)**	**0.043**
Prednisone, mg/day	−0.2 (−6–6)	0.94		
Hydroxychloroquine	158 (−27–344)	0.094	11 (−27–50)	0.57
Methotrexate	−26 (−64–13)	0.19	−38 (−102–25)	0.23
Mycophenolate mofetil	−9 (−49–32)	0.68		
Azathioprine	−18 (−49–14)	0.27		
Rituximab	−23 (−90–44)	0.50		
Belimumab	−51 (−118–16)	0.13	−30 (−118–57)	0.49
Subclinical carotid atherosclerosis				
cIMT, microns	**0.1 (0.01–0.2)**	**0.034**	0.07 (−0.1–0.3)	0.47
Carotid plaque	13 (−12–37)	0.32		

In this analysis, FGF23 is considered the dependent variable. BMI: body mass index; C: complement; CRP: C-reactive protein; LDL: low-density lipoprotein. ACA: anticardiolipin; cIMT: carotid intima-media thickness HDL: high-density lipoprotein; anti-ENAs: extractible nuclear antibodies. SLEDAI: Systemic Lupus Erythematosus Disease Activity Index. SLEDAI categories were defined as: 0, no activity; 1–5 mild; 6–10 moderate; >10 high activity, >20 very high activity. SLICC-DI: Systemic Lupus International Collaborating Clinics/American Colleague of Rheumatology Damage Index. Multivariable analysis is adjusted for age, smoking, hypertension, obesity, dyslipidemia, and vitamin D serum levels. Dyslipidemia is defined if total cholesterol >200 mg/d or triglycerides >150 mg/dL or LDL >130 mg/dL or HDL <40 mg/dL in males or HDL <50 mg/d in females. FGF23: fibroblast growth factor 23. Significant *p*-values are depicted in bold.

**Table 3:** Univariable relationship of individual disease score items to serum FGF23 levels.

			FGF23, pg/mL	
	n	%	Beta Coef. (95%)	*p*
*Katz index*				
History of cerebritis (seizure or organic brain syndrome)	12	6	**36 (4–69)**	**0.030**
History of pulmonary disease	10	5	−5 (−42–32)	0.80
Biopsy-proven diffuse proliferative glomerulonephritis	23	12	14 (−10–37)	0.26
4–6 ARA criteria for SLE satisfied to date	139	73	7 (−29–43)	0.70
7 or more ARA criteria for SLE satisfied to date	23	12	−6 (−30–17)	0.59
History of proteinuria (2+ or more)	62	32	−4 (−37–30)	0.83
Lowest recorded hematocrit to date = 30–37%	88	46	23 (−8–53)	0.14
Lowest recorded hematocrit to date < 30%	47	25	**−23 (−41–(−5))**	**0.014**
Highest recorded creatinine to date = 1.3–3	28	15	9 (−37–54)	0.71
Highest recorded creatinine to date > 3	3	2	35 (−24–95)	0.24
*SLEDAI*				
Seizures	1	0	124 (−62–310)	0.19
Psychosis	1	0	90 (−96–276)	0.34
Organic brain syndrome	0	0	-	-
Visual disturbance	1	0	−15 (−202–171)	0.87
Cranial nerve disorder	1	0	−43 (−230–143)	0.65
Lupus headache	1	0	126 (−60–312)	0.18
ACVA	0	0	-	-
Vasculitis	1	0	**271 (87–454)**	**<0.001**
Arthritis	9	3	−31 (−94–32)	0.33
Myositis	0	0	-	-
Urinary cylinders	7	3	16 (−56–87)	0.67
Hematuria	16	6	22 (−26–71)	0.36
Proteinuria	5	2	41 (−52–135)	0.39
Pyuria	11	4	27 (−31–84)	0.36
Rash	21	8	−12 (−54–31)	0.58
Alopecia	11	4	−10 (−67–48)	0.74
Mucosal ulcers	14	5	**−59 (−110–(−8))**	**0.023**
Pleurisy	3	1	42 (−90–175)	0.53
Pericarditis	1	0	43 (−144–230)	0.65
Low complement	76	28	6 (−21–32)	0.67
Elevated anti-DNA	85	31	−9 (−35–17)	0.50
Fever	2	1	−32 (−164–101)	0.64
Thrombopenia	10	4	5 (−62–72)	0.88
Leukopenia	19	7	15 (−30–61)	0.51
*SLICC-DI domains*				
Ocular	63	22	5 (−23–34)	0.71
Neuropsychiatric	40	14	23 (−11–57)	0.18
Renal	28	10	30 (−8–69)	0.12
Pulmonary	19	7	−9 (−61–42)	0.53
Cardiovascular	23	8	−20 (−60–21)	0.34
Peripheral vascular	34	12	−9 (−43–26)	0.62
Gastrointestinal manifestations	28	10	−14 (−52–24)	0.47
Musculoskeletal manifestations	89	31	**46 (22–70)**	**<0.001**
Skin	39	14	0.3 (−32–33)	0.98
Premature gonadal failure	19	7	32 (−13–78)	0.16
Diabetes (regardless of treatment)	18	6	8 (−39–55)	0.75
Malignancy (excluded dysplasia)	11	4	−20 (−81–40)	0.51

History of pulmonary disease refers to the presence of lupus pneumonitis, pulmonary hemorrhage, or pulmonary hypertension. SLEDAI: Systemic Lupus Erythematosus Disease Activity Index; SLE: Systemic Lupus Erythematosus. SLICC: Systemic Lupus International Collaborating Clinics/American Colleague of Rheumatology Damage Index. The presence of SLICC-DI domain involvement shown if points in the domain are ≥1. See Supplementary Table S1. ARA: American Rheumatism Association; ACVA: Acute Cerebrovascular Accident. FGF23: fibroblast growth factor 23. Significant *p*-values are depicted in bold.

## Data Availability

The data sets used and/or analyzed in the present study are available from the corresponding author upon request.

## Supplementary Materials

The following supporting information can be downloaded at: https://www.mdpi.com/article/10.3390/biom13081222/s1, Table S1: Relationship of SLICC-DI score items with FGF23.
