# Supplementary material for: Relationship of Fibroblast Growth Factor 23 Serum Levels with Disease Characteristics in Systemic Lupus Erythematosus Patients
Source: Biomolecules. 2023 Aug 5;13(8):1222. doi: 10.3390/biom13081222 (PMC10452311; doi:10.3390/biom13081222)
Supplement: Supplementary file 1 [file biomolecules-13-01222-s001.zip › biomolecules-2529367-supplementary.pdf]

biomolecules

MDPI

Supplementary Table S1. Relationship of SLICC-SDI score items with FGF23.

	n	%	FGF23, pg/ml Beta Coef. (95%)	p
**Ocular**				
Any cataract ever	29	11	−5 (−44–34)	0.81
Retinal change or optic atrophy	33	12	−24 (−61–14)	0.21
Points ≥1 in the domain	63	22	5 (−23–34)	0.71
Neuropsychiatric				
Cognitive impairment	7	3	10 (−62–81)	0.79
Seizures requiring therapy for 6 months	15	5	18 (−31–68)	0.47
Cerebrovascular accident ever	13	5	34 (−31–68)	0.27
Cranial or peripheral neuropathy	5	2	−27 (−121–68)	0.58
Transverse myelitis	1	0	-	
Points =>1 in the domain	40	14	23 (−11–57)	0.18
Renal				
Estimated or measured glomerular filtration rate <50%	13	5	5 (−52–62)	0.86
Proteinuria ≥3.5 g/24 h	7	3	**82 (12–152)**	**0.022**
End-stage renal disease	4	1	27 (−4–58)	0.090
Points ≥1 in the domain	28	10	30 (−8–69)	0.12
Pulmonary				
Pulmonary hypertension	4	1	−17 (−126–91)	0.75
Pulmonary fibrosis	4	1	15 (−80–109)	0.76
Shrinking lung	2	1	−3 (−136–130)	0.97
Pleural fibrosis	1	0	−50 (−237–138)	0.60
Pulmonary infarction	1	0	36 (−152–224)	0.71
Points ≥1 in the domain	19	7	−15 (−60–31)	0.53
Cardiovascular				
Angina or coronary artery bypass	4	1	−9 (−103–86)	0.85
Myocardial infarction ever	2	1	−30 (−163–103)	0.66
Cardiomyopathy	2	1	−43 (−176–90)	0.52
Valvular disease	9	3	−13 (−76–51)	0.70
Pericarditis for 6 months, or pericardiectomy	2	1	−13 (−146–120)	0.85
Points ≥1 in the domain	23	8	−20 (−60–21)	0.34
Peripheral vascular				
Claudication for 6 months	3	1	−13 (−121–95)	0.81
Minor tissue loss (pulp space)	5	2	6 (−78–90)	0.88
Significant tissue loss ever	0	0	-	-
Venous thrombosis	14	5	−30 (−81–22)	0.26
Points ≥1 in the domain	34	12	−9 (−43–26)	0.62
Gastrointestinal				

Infarction or resection of bowel	22	8	−14 (−56–29)	0.53
Mesenteric insufficiency	1	0	−67 (−254–121)	0.49
Infarction or resection of bowel below duodenum, spleen, liver, or chronic peritonitis	1	0	−29 (−217–158)	0.76
Stricture or upper gastrointestinal tract surgery ever	0	0	-	-
Pancreatic insufficiency	0	0	-	-
Points ≥1 in the domain	28	10	−14 (−52–24)	0.47
Musculoskeletal				
Muscle atrophy or weakness	3	1	62 (−47–170)	0.26
Deforming or erosive arthritis	40	15	**52 (20–84)**	**0.001**
Osteoporosis with fracture or vertebral collapse	23	9	**48 (6–91)**	**0.025**
Avascular necrosis	7	3	9 (−63–81)	0.80
Osteomyelitis	1	0	−17 (−204–171)	0.86
Tendon rupture	4	2	37 (−57–131)	0.44
Points ≥1 in the domain	89	31	**46 (22–70)**	**<0.001**
Skin				
Scarring chronic alopecia	16	6	−11 (−59–37)	0.66
Extensive scarring or panniculum	10	4	15 (−45–75)	0.62
Skin ulceration	4	1	−46 (−154–62)	0.40
Points ≥1 in the domain	39	14	0.3 (−32–33)	0.98
Premature gonadal failure	19	7	32 (−13–78)	0.16
Diabetes (regardless of treatment)	18	6	8 (−39–55)	0.75
Malignancy (exclude dysplasia)	11	4	−20 (−81–40)	0.51

SLICC items and domains represent the independent variable. SLICC: Systemic Lupus International Collaborating Clinics/American College of Rheumatology Damage Index. Significant p values are depicted in bold. FGF23: Fibroblast Growth Factor 23.
